# Island blues: indigenous knowledge of indigo-yielding plant species used by Hainan Miao and Li dyers on Hainan Island, China

**DOI:** 10.1186/s13002-019-0314-3

**Published:** 2019-07-03

**Authors:** Libin Zhang, Lu Wang, Anthony B. Cunningham, Yuru Shi, Yuhua Wang

**Affiliations:** 1 Department of Economic Plants and Biotechnology, Yunnan Key Laboratory for Wild Plant Resources, Kunming Institute of Botany, Chinese Academy of Sciences, 132# Lanhei Road, Kunming, 650201 China; 2 University of Chinese Academy of Sciences, Beijing, 100049 China; 3 School of Veterinary and Life Sciences, Murdoch University, 90 South St, Murdoch, WA 6150 Australia

**Keywords:** Indigo-yielding plant species, Indigo extraction methods, Ethnobotanical survey, Hainan Miao nationality, Li nationality, Hainan Island

## Abstract

**Background:**

Historically, indigo-yielding plant species were important cash crops from Central Asia to the southern United States and Central America. Indigo-dyed textiles were widely traded along the legendary Silk Road that linked China to Europe. Today, due to the labor-intensive nature of indigo extraction at the household level, lifestyle changes and the widespread availability of commercially produced indigo paste, traditional indigo extraction methods have declined in villages. Yet Li textile weavers on Hainan Island are internationally recognized as producers of indigo-dyed textile using warp ikat techniques. In contrast, Hainan Miao weavers produce indigo-dyed textiles using batik (wax resist) techniques. The aim of this study was to document the indigenous knowledge on indigo-yielding plant species used by both Hainan Miao and Li people on Hainan Island, China.

**Method:**

Ethnic uses were documented during three field surveys, through a questionnaire survey of 193 respondents, comprising 144 Hainan Miao and 49 Li traditional dyers. Mention index (QI), Availability index (AI), and Preference ranking (PR) of each indigo-yielding plant species were calculated to screen out plant resources with potential development value.

**Results:**

Five indigo-yielding plant species (from four plant families and four genera) were historically used by Hainan Miao and Li dyers. However, just four species are still in use. *Strobilanthes cusia* was the main indigo source for Hainan Miao dyers. Li dyers also commonly use *Indigofera species* (*I. tinctoria* and *I. suffruticosa*) for indigo extraction. *Wrightia laevis* is less commonly used as a contemporary indigo source. Indigo extraction by steeping in water to which lime is added to increase the pH is sharing by the five indigo-yielding plant species. *Strobilanthes cusia* had the highest QI, AI and PR values in Hainan Miao villages. *Indigofera tinctoria* had the highest QI and AI values, but *Indigofera suffruticosa* was preferred by Li dyers.

**Conclusion:**

In the process of modernization and urbanization, some Hainan Miao and Li dyers retain the traditional indigo extraction methods. We found that *Strobilanthes cusia* and *Indigofera tinctoria* have the most potential for sustainable indigo production in the future. Furthermore, this study documents the details of extraction method from *Wrightia laevis* for the first time and the use of *Ricinus communis* seeds in that process. As one of the last places globally where *Wrightia laevis* is still used for indigo production, the may also be a nice market among textile collectors and museums that keeps the tradition of *Wrightia laevis* production and use for indigo extraction alive.

## Background

Among all natural dyes, indigoids (such as indigo, Tyrian, and woad) are often regarded as the most important and the oldest dye used by mankind [1]. Indigo use dates back to at least 6000 years, it is referred to as ‘blue gold’ because of its great value of trading commodity [2, 3]. In addition to as natural dyes, indigo is also used as a food colorant and for medicinal use [2]. Historically, indigo is extracted from indigo-yielding plant species. The indigo-yielding plant species were important cash crops on farmers in India, China, Central America, South Carolina, the southern USA, and Indonesia [4, 5]. Indigo-dyed textiles were widely traded along the legendary Silk Road linking China to Europe [6]. However, in the twentieth century, most commercial natural indigo production declined after the advent of chemically synthetic indigo [2].

Nowadays, synthetic indigo still dominates denim dyeing with the consumption of several thousand tons annually [7]. However, the production of large-scale synthetic indigo presents a serious environmental issues. The synthetic indigo is produced by aniline. Aniline is derived from the petroleum product benzene. It is toxic and the synthesis involves hazardous chemicals [4]. On the contrary, traditional indigo dyes are of plant origin, which exhibits better bio-degradability and more sustainable than synthetic counterparts [8]. Recent years have seen a burgeoning interest in the natural dyes in textile research, eco-friendly fashion, and sustainable-production [4, 6]. Consequently, it is important to find potential indigo-yielding plant species for “greener,” eco-friendly indigo extraction at a larger scale.

One way to find the potential indigo-yielding plant species is to obtain the correct botanical provenance from the previous studies. For example, Cardon [9] recorded about 20 indigo-yielding plant species (7 families and 9 genera) used globally. Han’s study [10] showed that four indigo-yielding plant species (four families and four genera) were mainly used for indigo extraction in Ming (1368–1644 C.E.) and Qing Dynasties (1644–1911 C.E.) in China. However, for some indigo-yielding plant species, apart from a brief mention of use of indigo extraction, there are few contemporary accounts of the indigo extraction methods or how local dyers harvest in China. This knowledge gap needs to be filled before traditional indigo-yielding plant use and extraction methods disappear.

Therefore, the first step is to document the traditional knowledge of indigo-yielding plant species before it is lost. To document this, we chose Hainan Island as the study area. Firstly, Hainan Island is the main part of Hainan Province of China [11]. It is the largest island in the Indo-Burma Bio-diversity Hotspot and has the best-conserved tropical forest in China [12]. There are eight plant species that mentioned as blue dyestuff on Hainan Island [13]. Nevertheless, only one species (*Indigofera tinctoria* Linn.) was documented for indigo extraction [14]. Secondly, few studies [15, 16] in recent years indicated that the Hainan Miao and Li nationalities were still wearing indigo-dyed clothes during the traditional festivals and wedding.

**Fig. 1:**
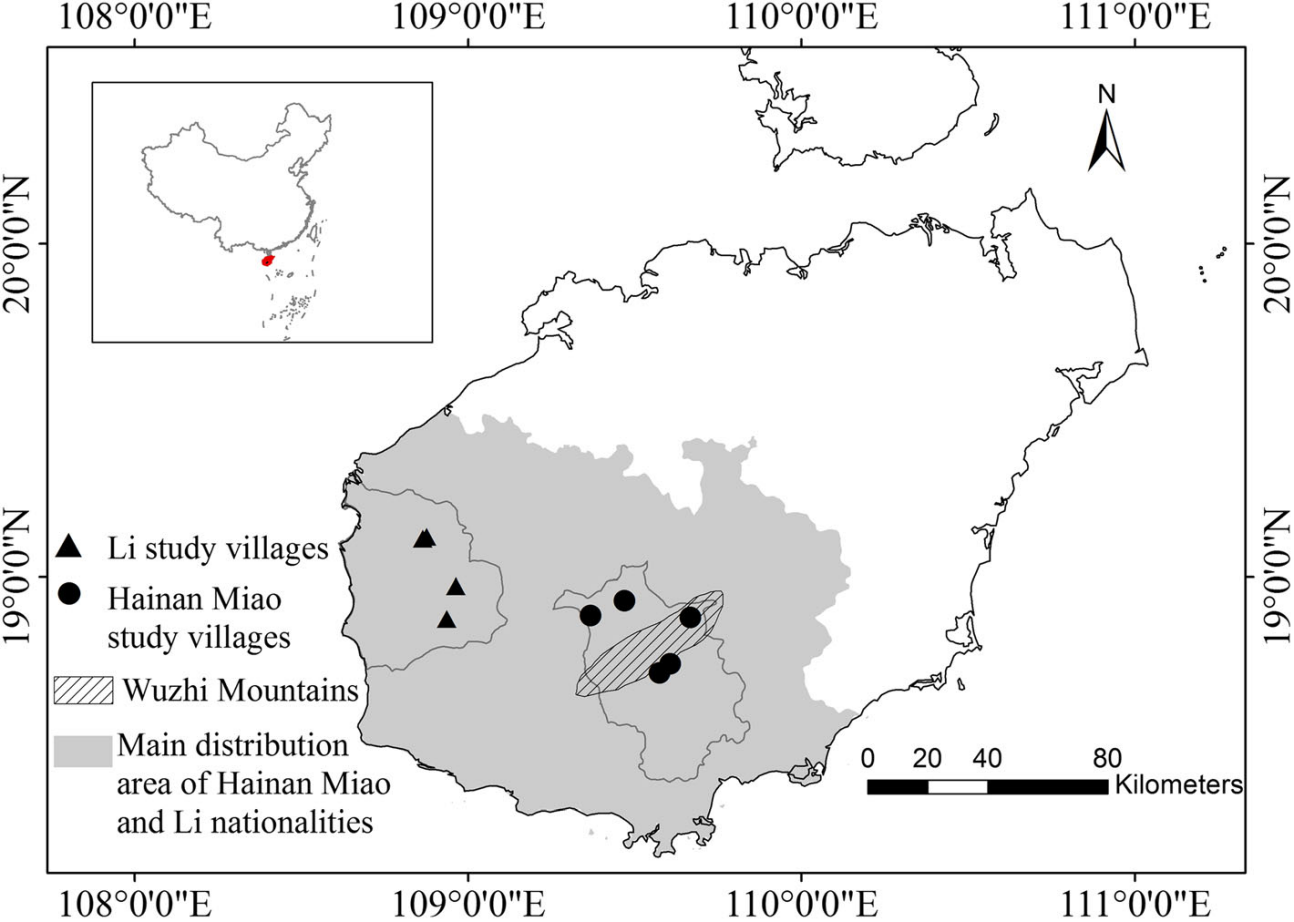
Location of the nine villages of Hainan Island in China selected as study sites (black dot, Hainan Miao study villages; black triangle, Li villages; slash region, Wuzhi Mountains; shaded region, mainly distribution area of Hainan Miao and Li nationalities)

Hainan Miao and Li are the two dominant minorities on Hainan Island [17]. They are mainly living in the south central part of Hainan Island [18] (Fig. 1). Hainan Miao (海南苗), also known as Kim Mun (a Mienic language in the border Hmong-Mien language group), immigrated from Guangxi Province about 600 years ago [17, 19]. Yet, the Li (黎) of Hainan Island, also known as Hlai (a Tai-Kadai language group), migrated from Guangdong and Guangxilong Province before the Qin Dynasty (221–206 B.C.) [17, 19]. Although the traditional costumes of the two minorities are indigo-dyed textile, Hainan Miao are expert of batik (wax resist) techniques while the Li textile weavers are famous of wrap ikat techniques [20, 21]. Prior research has focused on the indigo dyeing techniques and the meaning of the patterns [22, 23], but little attention has been paid to the scientific names of indigo-yielding species and detailed indigo extraction methods. Moreover, the traditional knowledge sharing of indigo-yielding plant species is very limited and passed on orally.

Therefore, the aim of this study is to document the indigenous knowledge of indigo-yielding species on Hainan Island, China.

## Methods

### Study site

The Hainan Miao villages are around Wuzhi Mountain area, and Li villages are all in Dongfang City on Hainan Island, China (Fig. 1). Prior information provided by local governments played instrumental roles in selecting research sites. The Wuzhi Mountains (18°38'~19°02' N; 109° 19'~109°44' E) are located in the hinterland of central Hainan Island. The Wuzhi Mountains are the source catchment for the three major rivers (Nandu, Changhua, and Wanquan) on Hainan Island, which are essential to the hydrology of the island [24]. The climate of the Wuzhi Mountains area is tropical monsoonal with an average yearly temperature of 25.7 °C [25]. Dongfang City (18°43'~19°18' N; 108° 36'~109°7' E) located in the southwest of Hainan Island, is the city with the longest sunshine time on Hainan Island [26]. The climate of Dongfang City is tropical monsoonal maritime climate with an annual average temperature of 23.5~24.5 °C; the average annual rainfall is unevenly distributed, with 1150 mm in the east and 950 mm in the west [27].

### Field survey and data collection

Two field surveys were performed in April and August in 2018, each of roughly 14 days. The third visit of 5 days was conducted in November 2018. Snowball sampling methods and questionnaires were employed to collect primary data [28]. Questionnaires for the collectors were shown in Table 1. Respondents, who were expert traditional dyers, were not forced to give their real names and answer all the questions. Prior informed consent [29] was taken verbally from all the respondents before documenting their traditional knowledge on various uses of indigo-yielding plant species. After getting permission, we took photos and videos of the main indigo extraction process.

**Table 1:** Questionnaire for the collectors

1 How many indigo yielding plant species do you use?	
2 What are their local names?	
3 What do their local names mean?	
4 From where do you collect indigo yielding plant species?	
5 Is there a lot of resources for indigo yielding plant species?	
6 Which species is the best material and why?	
7 During which months do you collect these indigo yielding species?	
8 How do you extract indigo from these indigo yielding species?	
9 How many lime powder do you use for extraction of indigo?	
10 How often do you extract indigo per year?	
11 How do you distinguish the quality of indigo paste?	
12 Can these plants in flowering period produce high quality?	
13 Has the plant species changed over the past couple of years?	
14 Why are you using these species for indigo extraction now?	

Preliminary work was done prior to the field surveys. We reviewed the *Flora of China* (Chinese vision)[30] and *Flora of Hainan* [31] and found the blue dye sources plant species distributed on Hainan Island. Then, we used photographs of them as a tool to assist the interview process [32]. During the field surveys, we asked the interpreters to help us translate the local language because none of the collectors could speak neither Hainan Miao nor Li language. Yet, the interpreters come from local villages. They spoke both Mandarin and local languages, so they could help to translate the local languages into Mandarin since some elder dyers could not speak fluent Mandarin.

### Statistical analysis

#### Demographic characteristics of the respondents

A total of 193 respondents (144 Hainan Miao and 49 Li traditional dyers) were selected. They are all women and represented six age groups (< 29, 30–39, 40–49, 50–59, 60–69, 70–79, 80–89 years). For Hainan Miao respondents, about one-third of participants (31.25%) were from 60 to 69 age group, less than 4.86% participants were at the 30 to 39 age group and nearly 13.19% were > 80 years older. For Li respondents, 36.73% of participants were from 60 to 69 age group, and 34.69% of participants were at the 50 to 59 age group. Nearly 13.19% were > 80 years old, and the number of respondents at the age of 30 to 39 is zero.

#### Quantitative analysis

In order to screen out potential plant resources for making “greener” and more eco-friendly indigo extraction methods in the future, we used questions 1, 4, 5, and 6 above to calculate the mention indices (QI), availability indices (AI), and preference rankings (PR) of each indigo-yielding plant species. QI was to test homogeneity of knowledge and QI =number of mentions/number of informants [33]. AI was to evaluate the resources and accessibility of indigo-yielding plant species, and the details of the calculation method are seen in Table 2 [34]. PR ranged from 1 to 5, and all informants were oriented on each species and asked to mark the highest value (5) for most preferred and the lowest value (1) for the least preferred [35]. In order to compare the indigenous knowledge change of indigo-yielding plant species, we calculated QI and AI values of indigo-yielding plant species used in the past and now separately. Besides, the final value of each species is the average of all informant responses. All the botanical names used conform to accepted names in “The Plant List” [36].

**Table 2:** QI (mention index), AI (availability index), and AI correction index

Index	Answer	Value
QI	Not mentioned	0
	Mentioned	1
AI	Never seen	0
	Occasionally seen	1
	Often seen	2
	Very common	3
Correction index	Somewhere	− 1
	Some places	− 0.5
	Everywhere	0

## Results

### Indigenous knowledge of indigo-yielding plant species

In this study, five plant species [*Strobilanthes cusia* (Nees) Kuntze, *Wrightia laevis* Hook. F, *Indigofera suffruticosa* Mill., *Indigofera tinctoria* Linn., and *Persicaria tinctoria* (Aiton) H.Gross] were documented, but *Persicaria tinctoria* is no longer used and even its seeds are not preserved (Table 3). Interestingly, there is under-differentiation of the two species (*Indigofera suffruticosa* and *Indigofera tinctoria*), which are given the same vernacular name in the study sites. Both of these *Indigofera* species were called *gam za/ging* by the Hainan Miao and *be fa* by the Li dyers. This under-differentiation occurs despite both Hainan Miao and Li dyers being able to distinguish these two plant species on the basis of the different fruit shapes, leaf sizes, and heights of these *Indigofera* species. Local dyers observed that *Indigofera tinctoria* has straight fruits, smaller leaves and is around a meter high, while *Indigofera suffruticosa* has curved fruits, its leaves are bigger, and the plant can grow into a tall bush over 2 m high. However, *Strobilanthes cusia* was given two vernacular names by Hainan Miao dyers according to the leaf sizes. Those with large leaves that grow under the shadow of trees were called *gam luo*, while the small ones were called *gam dun*. *Luo* means large and *dun* means small in local Hainan Miao language.

**Table 3:** Indigo-yielding plant species used by Hainan Miao and Li dyers on Hainan Island, China (species are listed alphabetically)

Family	Scientific name	Chinese character	Local name		Habitat	QI_1_		QI_2_		AI_1_		AI_2_		PR		Voucher number
			M	L		M	L	M	L	M	L	M	L	M	L	
Acanthaceae	*Strobilanthes cusia* (Nees) Kuntze	板蓝	*gam dun; gam luo*	*be fa ban*;*be fa ao*	Semi-wild and cultivated	1	0.21	0.97	0.04	1.8	1.5	1.17	0.75	4.88	3.10	ZLB03 ZLB05-09 ZLB11 ZLB47
Apocynaceae	*Wrightia laevis* Hook.f.	蓝树	*dian gam*	/	Cultivated	1	/	0.25	/	1.48	/	1.01	/	2.95	/	ZLB17-20 ZLB60
Leguminosae	*Indigofera suffruticosa* Mill	野青树	*gam za, gang ging*	*be fa*	Cultivated	0.94	0.9	0.19	0.19	1.45	1.71	0.66	1.05	4.09	4.88	ZLB12 ZLB14-16 ZLB41
Leguminosae	*Indigofera tinctoria* L.	木蓝	*gam za, gam ging*	*be fa*	Cultivated	0.83	1	0.08	0.63	1.43	1.78	0.58	1.47	3.86	4.42	ZLB13 ZLB043 ZLB50 ZLB65
Polygonaceae	*Persicaria tinctoria* (Aiton) H. Gross	蓼蓝	*da gam, gam liao*	/	Cultivated	0.22	/	0	/	1.47	/	0	/	3.13	/	Null(Do not use anymore)

Note: *QI*_*1*_ mention index of indigo-yielding plant species used in the past, *QI*_*2*_ mentioned index of indigo-yielding plant species used in the present, *AI*_*1*_ availability index of indigo-yielding plant species used in the past, *AI*_*2*_ availability index of indigo-yielding plant species used in the present, *PR* preference ranking, *M* Hainan Miao nationality, *L* Li nationality

For the indigo-yielding plant species used in the past, *Strobilanthes cusia* had the highest QI value (1.0) and AI value (1.8) among Hainan Miao dyers. However, things are different in Li villages that *Indigofera tinctoria* had the highest QI value (1.0) and AI value (1.8). It implied that *Indigofera tinctoria* is the most commonly used and the most accessible resource of indigo paste for Li dyers. For the indigo-yielding plant species used in the present, in the Hainan Miao study villages, *Strobilanthes cusia* is the main source of indigo, while *Indigofera suffruticosa*, *Indigofera tinctoria*, and *Wrightia laevis* were used as supplements for indigo extraction. *Strobilanthes cusia* still had the highest QI value (0.97) and AI value (1.17) among Hainan Miao dyers and *Indigofera tinctoria* still had the highest QI value (0.63) and AI value (1.47) for Li dyers. However, both indices values have a downward trend of all five indigo-yielding plant species especially the QI and AI value of *Persicaria tinctoria* are zero because Hainan Miao dyers no longer use this species.

As for PR values, Hainan Miao and Li respondents preferred the species that could produce a good quality indigo paste. Based on the faith that those with dark blue and reddish indigo paste were of good quality, Hainan Miao dyers ranked the highest mark to *Strobilanthes cusia* (4.88), while *Wrightia laevis* (2.95) was the least favored. Li dyers believed that *Indigofera suffruticosa* (4.88) was preferred according to their experience. Further, it is interesting that *Strobilanthes cusia* was given the lowest score (3.10) by Li dyers, which was very different from the preference of Hainan Miao dyers. This can be explained by the different environmental conditions of the study sites. The Li study villages are at a lower altitude and are drier, so are less suitable for the growth of *Strobilanthes cusia*. Only about 20% (*n* = 10) Li dyers knew how to use *Strobilanthes cusia*, and this is known through gift exchange. This occurred when Li people who lived near mountain areas brought *Strobilanthes cusia* as a gift when visiting their relatives.

### Indigo extraction methods

**Fig. 2:**
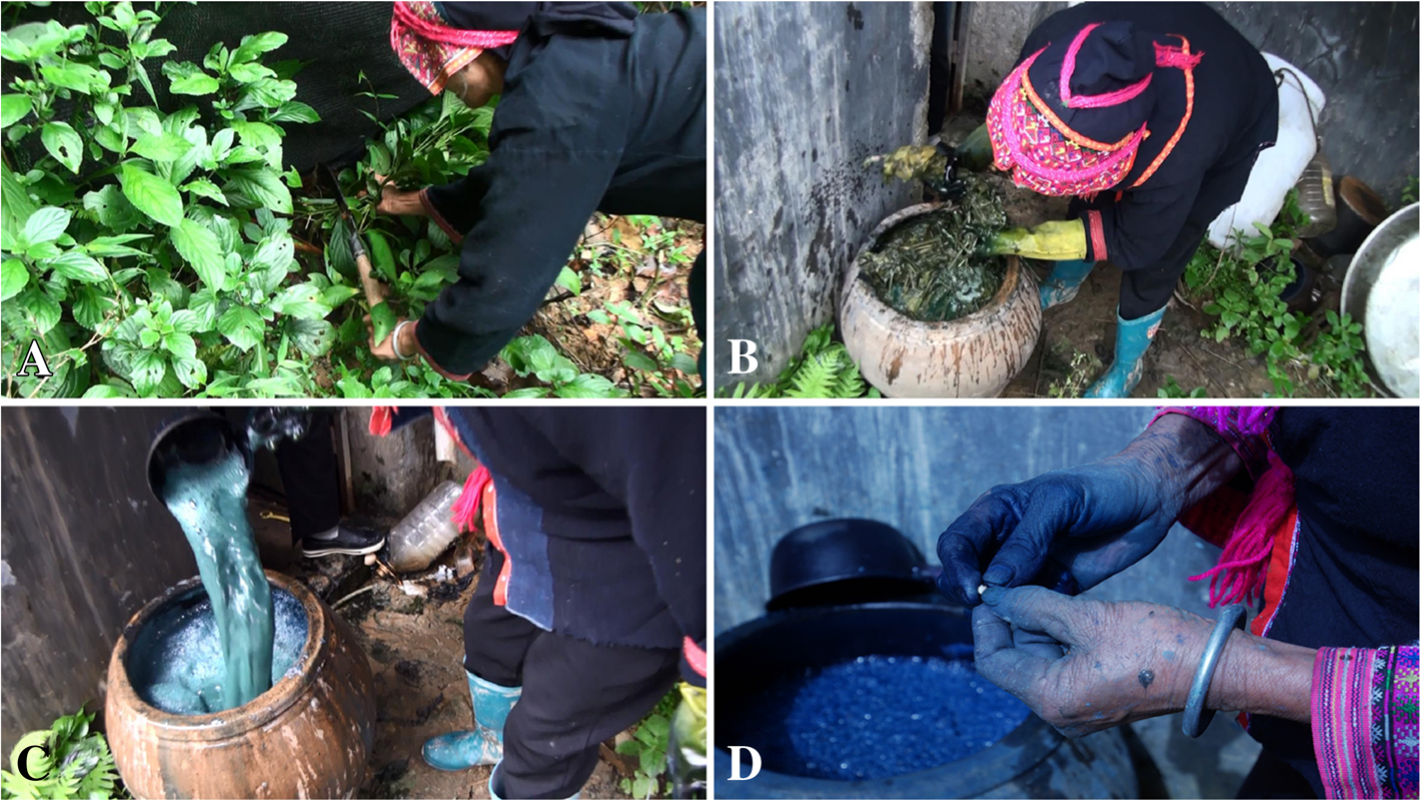
Indigo extraction method of *Strobilanthes cusia* by Hainan Miao dyers. **a** *Strobilanthes cusia* harvesting in the home garden. **b** Removing the rotten aerial parts from the the extraction vat. **c** Using plastic scoop for aeration. **d** *Ricinus communis* seed use

**Fig. 3:**
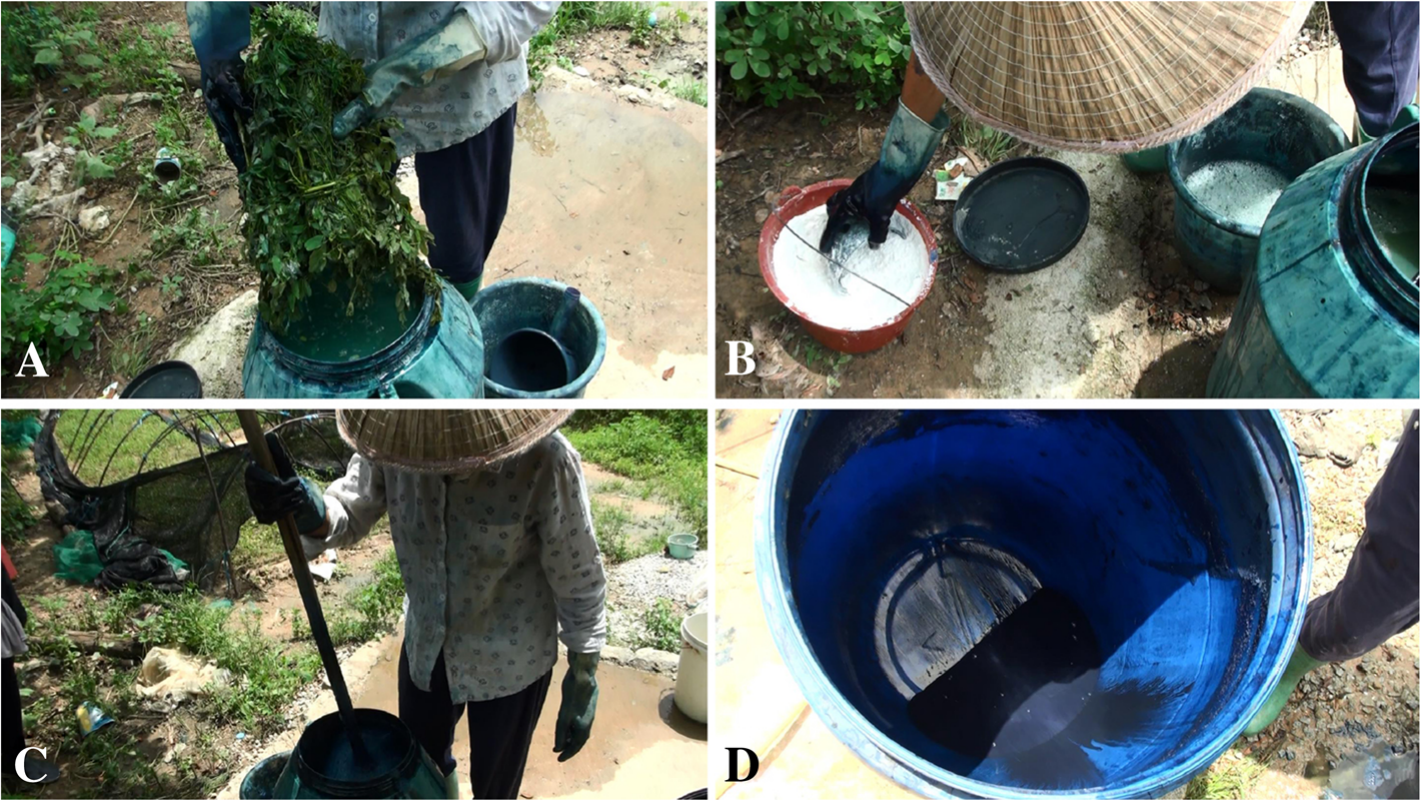
Indigo extraction method of *Indigofera tinctoria* by Li dyers. **a** Removing the rotten aerial parts from the the extraction vat. **b** Filtering the lime powder. **c** Agitation for oxygenation. **d** Sedimentation on the bottom

Although Hainan Miao and Li dyers on Hainan Island traditionally used indigo-yielding plant species for centuries, details of the indigo extraction processes have not been thoroughly documented. We found that both Hainan Miao and Li dyers use the steeping method for five indigo-yielding plant species. The indigo extraction method was shown in Figs. 2 and 3: (1) harvesting: the fresh materials of indigo-yielding plant species are harvested and transported from the field to their homes; (2) fermentation: the materials were bent and left to steep in a cistern and added cool water to completely immerse the materials. Then removed the materials from the fermentation vat when they rotted. This process usually took 2 to 3 days depending on the amount of soaking materials and the weather condition, up to 1 week; (3) oxygenation: lime powder water was added to the fermentation vat, and the mixture was vigorously stirred for 0.5 to 1.5 h. When the foam was less and the foam color was blue and reddish, the dyers stop the oxygenation process. (4) Sedimentation: after resting for one or two nights, the blue sediment was on the bottom of the cistern and was called *lan dian* (蓝靛) (translation: indigo paste). The *lan dian*, compacted in paste, will transfer to a small plastics bucket or pot to keep it moist for storage. Moreover, the five indigo-yielding plant species could be mixed in practical production according to the information of the questionnaire.

The main indigo extraction process is almost the same between Hainan Miao and Li dyers, but there are still some differences in teams of indigo-yielding plant species, part used, harvest season, fermentation duration, amount of lime, oxygenation method tools, and annual production frequency (Table 4). What is most interesting is the *Ricinus communis* seed use in five Hainan Miao study villages. *Ricinus communis* L. (Euphorbiaceae), known as *gen zong* in Hainan Miao language, has two varieties that are distinguished by the color of their stems and leaves (voucher numbers ZLB45, ZLB60). A small number of *Ricinus communis* seeds were added to the extraction vat at the end of the oxygenation process to help reduce the foam. This is because the Hainan Miao dyers believed that the foam reduced the quality of indigo paste. Commonly 2–5 still moist seeds were crushed on the side of the extraction vat. Some dyers stripped off the seed coat before crushing them. The *Ricinus communis* seeds were then held while stirring in the extraction vat for 1–2 min before throwing away the seeds at the end of this process. From either variety were used, with no difference in relation to the *Ricinus communis* variety according to the Hainan Miao dyers.

**Table 4:** Comparison of the indigo extraction methods between Hainan Miao and Li dyers on Hainan Island, China

	Indigo-yielding species	Part used	Harvest season	Fermentation duration/h	Amount of lime	Oxygenation method tools	Production frequency/year
Li	*Strobilanthes cusia* (Neek) Kuntze	Leaves and stems	All seasons (20%); July–October (80%)	24 (90%); 24–48 (10%)	450g (10%); 250–500 g (80%); 500–1000 g (10%)	Homemade bamboo tools	Once (10%); twice (47%); once to twice (33%)
	*Indigofera suffruticosa* Mill.	Leaves and stems and ripe fruit	July–September (95%); October–November (5%)	24 (95%); 24–48 (5%)			
	*Indigofera tinctoria* L.	Leaves and stems and ripe fruit	July–September (91%); October–November (9%)	24 (15%); 24–48 (85%)			
Hainan Miao	*Strobilanthes cusia (Neek)* Kuntze	Leaves and stems	All seasons (50%); July–December (48%); November–January (2%)	48–72 (22%); 72–96 (70%); 72–168 (8%)	250 g (17%); 250–500 g (59%); 200–500 g (24%)	Plastic scoop	Once (44%); once to twice (33%); twice to three times (23%)
	*Indigofera suffruticosa* Mill.	Leaves and stems		24–48 (10%); 48–72 (70%); 72–120 (20%)			
	*Indigofera tinctoria* L.	Leaves and stems		24–48 (14%); 48–72 (73%); 72–120 (11%)			
	*Persicaria tinctoria* (Aiton) H.Gross	Leaves and stems		24–72 (69%); 72–96 (19%); 72–168 (12%)			
	*Wrightia laevis* Hook.f.	Leaves and young shoots		24–72 (36%); 72–120 (45%); 72–168 (19%)			

Note: Percentages represent the proportion of the total number of the informants referring to these values

## Discussion

### Indigo-yielding plant species in use

In the previous study [9], few brief mentions of *Wrightia laevis* use for indigo but no detail is given in any of these accounts of how local dyers harvest. In this study, the detailed indigo extraction method of *Wrightia laevis* was first documented (Table 4). Although *Wrightia laevis* was the least favored (PR = 2.95) for its low indigo paste quality, it was still as a supplement because this species is easy to find around the villages (*A*_1_ = 1.48, *A*_2_ = 1.01). For Hainan Miao dyers, the most cited and favored species is *Strobilanthes cusia.* The respondents gave the reason that *Strobilanthes cusia* provide the highest yield of indigo paste according to their experience, which is consistent with Chanayath’s result that *Strobilanthes cusia* gave more indigo than *Indigofera tinctoria* (the main indigo source in the world) in the ratio of 4:3 [37]. Further, it is easy to survive by cuttings and become the main source of indigo paste in Hainan Miao villages. In China, apart from the indigo paste source, *Strobilanthes cusia* is also a frequently used Chinese herbal medicine (TMC) that recorded in “People’s Republic of China Pharmacopoeia 2015” [38]. For example, its roots are known as “Nan-Ban-Lan-Gen (南板蓝根),” commonly used to prevent and treat virus-related respiratory diseases such as influenza virus infection [39]. Its leaves and stems are produced as “indigo naturalis (青黛)” to treat chronic diseases such as psoriasis [40]. During the outbreak of severe acute respiratory syndrome (SARS) in 2003, *Strobilanthes cusia* has been listed as one of the eight major anti-SARS medicines [41, 42]. Moreover, prior ethnobotanical surveys have shown that the tender stems and leaves of this species are also as an edible vegetable in Xishuangbanna, Yunnan Province, China [43]. So it is not difficult to find that *Strobilanthes cusia* has potential commercial value for sustainable indigo production and medicinal use.

For Li traditional dyers, the most commonly used indigo-yielding species is *Indigofera tinctoria*, while the most preferred (4.88) one is *Indigofera suffruticosa*. This is because the growth period of *Indigofera suffruticosa* is longer than *Indigofera tinctoria*. So the acreage of *Indigofera suffruticosa* is smaller in Li villages in spite of its high indigo quality. *Indigofera tinctoria*, commonly known as “true indigo,” is the most widely exploited of all indigo-yielding plant species [44]. This tropical species is thought to “have spread with dyeing technology (and the word *nila* itself, as already noted) from India first to South East Asia, then through the Middle East to parts of Africa, and later to America” [45]. Consequently, *Indigofera tinctoria* is another potential candidate for sustainable indigo production.

### The characteristics of the indigo extraction methods on Hainan Island

The steeping methods we documented on Hainan Island were also reported in Indian, Japan and the south of China [9, 46–48]. However, the steeping method practiced by Hainan Miao and Li dyers differs from those used by other minorities. For example, the parts used of *Indigofera tinctoria* and *Indigofera suffruticosa* were different. A previous study [1] showed that the indigo compounds from the two species are extracted by steeping leaves, but we found the stems, leaves, and ripe fruit were used in Li villages. This is because Li dyers believed the high-quality indigo paste needed the fruit ripening period according to the experience. So, they usually sow in March to April and harvest from July to September.

In addition, the use of a few *Ricinus communis* seeds after the oxygenation process is first documented in this study. The *Ricinus communis* seeds were used at the end of the oxygenation process by Hainan Miao dyers but Li dyers did not use them. This is because Hainan Miao believed that *Ricinus communis* seed use could reduce foam to improve indigo paste quality. This introduced plant species are commonly found in all five Hainan Miao villages, which might be one reason for its use. However, the mechanism of *Ricinus communis* seed use to improve indigo paste quality is still unknown. Legrand [49] described another plant species used in the steeping method in Santiago Niltepec of Mexico, the pulpy fruit of a local plant known as “*gulavere*” is added to the oxygenation vat to help accelerate sedimentation. Nevertheless, there is no information on which species the local name *gulavere* represents or on its possible chemical function in assisting indigo sedimentation.

### Chemical pigments formation of indigo extraction

Indican (indoxyl-3-O-β-D-glucoside), a second plant metabolite, is the most prominent indigo precursor [50]. Stored in the vacuole of indigo-yielding plant species, indican was hydrolyzed and produce indoxyl after steeping in the water, and thereby indoxyl molecules produce a chemical reaction to form indigo pigment [51]. In most common situations, indigo is blue color and indirubin is red [52]. If there were fewer impurities in the indigo paste, the blue and red color would be more obvious. Namely, to a certain extent, the color implies the purity of chemical pigments of indigo paste. This may explain the folk knowledge that Hainan Miao and Li respondents believed those with dark blue and reddish indigo paste were of good quality.

Production of indigo paste at the village level has some disadvantages compared to synthetic indigo for two main reasons. Firstly, in addition to water, the actual indigo content in the indigo paste could vary from 2 to 70%, the rest were lime, indirubin, and other organic materials [47]. Yet, synthetic indigo owns consistently high purity, which has always exceeded 90% [1]. To improve the indigo purity of traditional indigo-extracting methods, more attention should be paid to the indican metabolism since the indigo pigments come from the precursor indican as discussed above. Secondly, the method of synthetic indigo production is controllable because it follows specific chemical reactions [6]. However, the indigo yield of the traditional method is so hard to control that even a senior dyer cannot guarantee the same indigo yield every time. The water-to-biomass ratio, fermentation duration, fermentation temperature, lime quality, pH, and dissolved oxygen concentration are directly or indirectly related to indigo yield [11]. Therefore, a better understanding of these physicochemical parameters may contribute to adopt novel technologies to help standardize the traditional indigo extraction methods.

## Conclusion

Historically, the traditional use of indigo as a pigment took place across many societies because of its high value as a trading commodity. Despite the process of modernization and urbanization, some Hainan Miao and Li dyers have still maintained the traditional indigo extraction methods. We found that *Strobilanthes cusia* and *Indigofera tinctoria* are the best potential candidates for sustainable indigo production. In addition, the detailed use of *Wrightia laevis* as an indigo source and *Ricinus communis* seed use during oxygenation were first documented in this study. More attention should be given to adopting novel technologies to make natural indigo as a compatible and sustainable alternative to synthetic colorants.

## Data Availability

The datasets used and/or analyzed during the current study are available from the corresponding author on reasonable request.

## Abbreviations

AI: Availability index
PR: Preference ranking
QI: Mention index
